# 1176. Assessing the Impact of Collaborative Antimicrobial Stewardship Guidelines on the Use of Antibiotics in Hospitalized Veterans with COVID-19 from Five Veterans Health Administration (VHA) Hospitals

**DOI:** 10.1093/ofid/ofad500.1016

**Published:** 2023-11-27

**Authors:** Neena Thomas-Gosain, Kelly W Davis, Sage Hendrickson, Todd Hulgan, Parmida Parvaz, Caroline Powers, John H Read, Milner Staub, Jessica G Bennett

**Affiliations:** University of Tennessee Health Science Center, Memphis, Tennessee; Lexington VA HealthCare System, Lexington, Kentucky; Tennessee Valley Veterans Affairs Healthcare System, Nashville, Tennessee; Tennessee Valley Healthcare System and Vanderbilt University Medical Center, Nashville, Tennessee; Tennessee Valley Healthcare System, Nashville, Tennessee; VA St. Louis Health Care System, St. Louis, Missouri; University of Tennessee Health Science Center College of Medicine, Memphis, Tennessee; Vanderbilt University Medical Center and VA Tennessee Valley Healthcare System, Nashville, Tennessee; VAMC Memphis, Germantown, Tennessee

## Abstract

**Background:**

Limited data and resources drove collaboration among healthcare providers treating COVID-19. In July 2021 a multi-disciplinary committee representing all facilities in Veterans Integrated Service Network (VISN) 9 developed COVID-19 guidelines for bacterial co-infection in hospitalized COVID-19 patients. We assessed the impact of these guidelines on appropriate antibiotic utilization (AU) in admissions for COVID-19.

**Methods:**

AU in COVID-19 admissions to VISN 9 facilities from 08/01/20 to 08/31/21 (pre-guidelines) were reviewed and compared to 11/01/21 to 11/30/22 (post-guidelines). COVID-19 positivity, hospitalization, and AU data were pulled from the Corporate Data Warehouse. COVID-19-specific antibiotic stewardship (AS) interventions and guideline distribution plans were collected for each facility. A weighted random sample of patients from each facility who received antibiotics was chart reviewed for appropriateness of AU. AU for non-respiratory indications were excluded from analysis (Table 1). AU was considered appropriate if at least one guideline criteria was met (Table 2). Antibiotic appropriateness was compared before and after guideline distribution for each site using t-test. The odds of appropriate antibiotic prescribing in the VISN after guidance distribution was determined by logistic regression adjusted for site.

**Figure:**
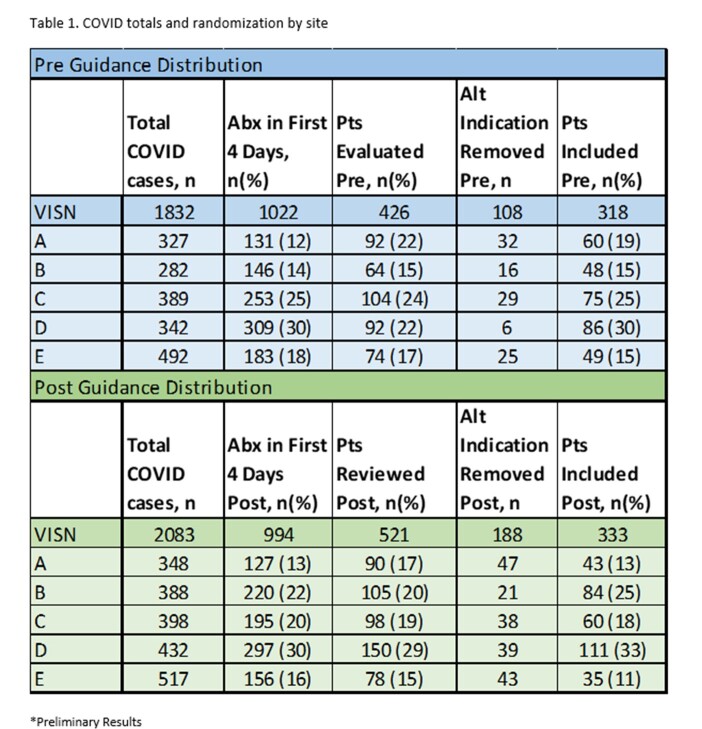

**Figure:**
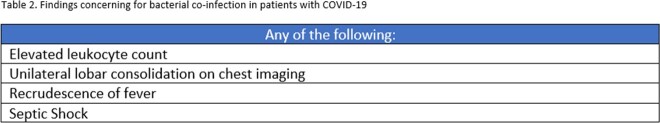

**Results:**

Initial rates of antibiotic use varied among facilities both pre and post guideline distribution (Table 1). Facility-specific interventions and guideline distribution also varied (Table 3). There was no significant change in the frequency of appropriateness for any individual site. Similarly, the odds of AU appropriateness for the VISN was not significantly changed in the post-guideline period (p=0.7) (Table 4).

**Figure:**
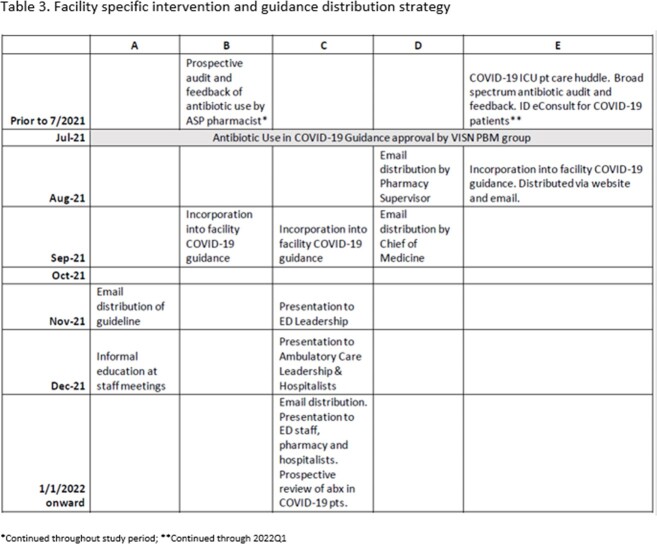

**Figure:**
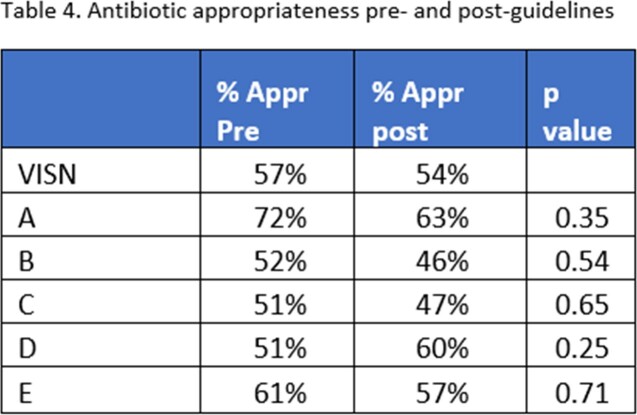

**Conclusion:**

AU appropriateness did not change significantly after guidance distribution, likely due to a myriad of reasons. Reprioritization of AS programs’ focus in the post-guidance period may have shifted resources away from initiatives targeting AU in COVID-19 patients. Additionally, guidance was distributed mid-2021 potentially missing the window for largest impact. Collaboration can fill in gaps for many ASPs; but the success of any intervention is multifactorial.

**Disclosures:**

**Milner Staub, MD, MPH**, Gilead: Stocks/Bonds|Johnson & Johnson: Stocks/Bonds

